# Secure State Estimation for Motion Monitoring of Intelligent Connected Vehicle Systems

**DOI:** 10.3390/s20051253

**Published:** 2020-02-25

**Authors:** Xiulan Song, Xiaoxin Lou, Junwei Zhu, Defeng He

**Affiliations:** College of Information Engineering, Zhejiang University of Technology, Hangzhou 310023, China; lxx@zjut.edu.cn (X.L.); junweizhu1001@zjut.edu.cn (J.Z.); hdfzj@zjut.edu.cn (D.H.)

**Keywords:** connected vehicles, cooperative adaptive cruise control, state estimation, remote monitoring, cyber-attack

## Abstract

This paper considers the state estimation problem of intelligent connected vehicle systems under the false data injection attack in wireless monitoring networks. We propose a new secure state estimation method to reconstruct the motion states of the connected vehicles equipped with cooperative adaptive cruise control (CACC) systems. First, the set of CACC models combined with Proportion-Differentiation (PD) controllers are used to represent the longitudinal dynamics of the intelligent connected vehicle systems. Then the notion of sparseness is employed to model the false data injection attack of the wireless networks of the monitoring platform. According to the corrupted data of the vehicles’ states, the compressed sensing principle is used to describe the secure state estimation problem of the connected vehicles. Moreover, the *L*_1_ norm optimization problem is solved to reconstruct the motion states of the vehicles based on the orthogonaldecomposition. Finally, the simulation experiments verify that the proposed method can effectively reconstruct the motion states of vehicles for remote monitoring of the intelligent connected vehicle system.

## 1. Introduction

With the rapid increase in the number of road vehicles, the problems of traffic congestion, exhaust emissions and safety are becoming more and more serious in big cities and/or urban areas [1,2]. Intelligent transportation systems (ITS) technology is one of the potential solutions to lessen these problems [3,4,5,6]. Benefiting from the development of the wireless communication technology, the intelligent connected vehicle system is one of such ITS that can potentially reduce the risk of accidents and increase traffic throughput by resorting the Internet of Vehicles (IoV), e.g., vehicle-to-vehicle (V2V), vehicle-to-infrastructure (V2I), vehicle-to-road (V2R) communication, etc. However, due to the open nature of wireless communication and the high-mobility of moving vehicles, the IoV communication networks are vulnerable to packet dropping, communication time-delay and malicious cyber-attack [7]. Especially, the malicious cyber-attack, e.g., false data injection attack, will cause mistakes in the decision makers of ITS that may lead to serious traffic accidents [7]. Hence, it is necessary to realize the secure estimation of vehicle motion states in the remote monitoring platform of intelligent connected vehicle systems.

The intelligent connected vehicle system contains many on-board sensors, controllers, actuators and other units, and integrates modern wireless communication and network technologies. It can realize intelligent information exchange between moving vehicles and X (i.e., cars, roads, people, clouds, etc.) and can real-time sense the complex surroundings. Based on IoV and intelligent sensing, the intelligent connected vehicle system makes intelligent decisions in real time to help drivers to achieve collaborative control of a group of connected vehicles, and ultimately achieve automated intelligent driving with safety, efficiency, ride comfort and energy-saving [5,6,7,8]. However, there are some inherent weakness in wireless IoV, such as communication delays and packet dropping. Moreover, because of the openness of wireless networks, there may be artificial attacks in intelligent connected vehicle systems [9,10,11,12,13]. In the past decades, many efforts have been directed at the study of the issues of wireless networked control of connected vehicle systems and rich control methods to compensate network defects such as packet dropping and communication delay have been proposed. For example, Ploeg et al. [14] and [15] proposed the *L_p_* norm-based string stabilizing control method and discussed the string stability of cooperative adaptive cruise control (CACC) systems in unreliable communication networks. Ploeg et al. [16] considered the communication delay problem of IoV and achieved the stability of the intelligent connected vehicle systems using CACC approaches [12,13]. Moreover, when communication delay and packet loss occur simultaneously, the CACC system of connected vehicles will actively degrade to the traditional adaptive cruise control (ACC) system [5] while ensuring string stability that is better than the one of the ACC system [17].

In recent years, the cyber-security issue has increasingly gained attention in the automotive and academic communities due to widely used wireless communication networks of IoV and very dangerous results caused by cyber-attacks. For example, in July 2015, the "white hat hackers" Miller and Wallacek demonstrated how to "hijack" remote command methods by invading Chrysler Uconnect vehicle systems when driving, and eventually caused a "roll over" [8]. This remote cyber-attack event has made many scholars investigate the cyber-security problem in the field of intelligent connected vehicle system with various embedded CACC systems. For instance, Biron et al. [18] proposed a sliding mode observer algorithm for detecting the occurrence of denial of service (DOS) attacks in the networks and estimating the magnitude of the delay. Amoonzadeh et al. [19] studied the effects of the tampered sensors, which seriously affects the string stability of connected vehicle platooning systems. Dadras et al. [20] studied the ability of an attacker to invade a networked vehicle by remote attack and showed that attackers can remotely control the individual position and speed of networked vehicles. Liu et al. [21] showed the serious impacts of the cyber-attack on automated platoon systems and proposed a design approach for safe platooning controllers. Following the method in [21], the safe inter-vehicle distance is greatly shortened. Alipour-Fanid et al. [22] conducted a comprehensive analysis of stability and safety for vehicle strings over wireless Rician fading channels under jamming attacks. They showed that fading channels degrade the performance of CACC systems through rich simulation experiments under various attacked scenarios. In addition, Li et al. [23] summerized the influences of cyber-attacks on longitudinal safety of connected and automated vehicles via extensive simulations and sensitivity analysis.

Due to the significant threat of cyber-attacks to the safety of persons and property, in the automotive and academic communities more and more scholars have studied the safety and security problems of connected and networked vehicles under cyber-attack. For example, Massoumnia et al. [24] and Blanke et al. [25] proposed the residual test method to detect the false data injection attack for networked systems including connected and networked vehicles. Since each measured value has a residual signal, the measured value is considered to be attacked if the residual value is greater than a given threshold. However, if an attacker sets the special data so that the residual is still less than the threshold, this method cannot be applied well. Another method to again cyber-attack is to use the idea of robust control. It can achieve stability of uncertain systems when the system is destroyed by some unknown disturbances. However, in this method perturbations are assumed to be bounded by some ranges [26]. For instance, Schenato et al. [27] considered the disturbance as a certain random process if the wireless channel is interfered and analyzed the control and estimation problems of networked control systems. Lately, Lu and Yang [28] designed a Luenberger-like observer and used a new projection operator method to reconstruct the states from a series of continuous measurements of cyber-physical systems. Wu et al. [29] proposed a sliding mode observer for estimating the system states from the measurement data of contaminated sensors. Fawzi et al. [30] and Hwan et al. [31] assumed that the attacked states satisfied sparseness and then proposed the use of *L*_1_ norm optimization to reconstruct the states of cyber-physical systems including connected vehicle systems.

Aiming at the problem of secure state estimation of intelligent connected vehicle systems under the attack of false data injection in the wireless monitoring networks, this paper proposes a secure state estimation method to reconstruct the motion states of the connected and networked vehicles equipped with CACC systems. The main idea of the method is to use the principle of compressed sensing based on the notion of sparseness. By adopting Proportion-Differentiation (PD) controllers, e.g., [12,13], the set of CACC models is used to represent the longitudinal dynamics of the intelligent connected vehicle systems. Due to adversarial attack to the intelligent connected vehicle system, the number of attacked sensors is assumed to be less than the half of the total sensors. Then the attacked vector can be regarded as a sparse vector and transformed into an *L*_1_ norm optimization problem for secure state reconstruction. Finally, the simulation experiments verify that the proposed method can effectively reconstruct the motion states of vehicles for remote monitoring of the intelligent connected vehicle system.

The remaining of this paper is organized as follows: In Section 2, the set of CACC models and false data injection attack models are formulated. In Section 3, we present the secure state estimation approach and verify the applicability of the approach. Then we demonstrate the proposed approach through some classical simulations in Section 4. Finally, we conclude the paper in Section 5.

## 2. Problem Description

To increase the efficiency of ITS, road vehicles are generally arranged into a vehicle platoon to reduce the risk of accidents and increase traffic throughput. Here a group of vehicles is assumed to be controlled by some stabilizing CACC systems to form a vehicle platoon with guaranteed string stability. From the department of transportation, it is necessary to remotely monitor the real motion states of each vehicle in the connected platoon system by the monitoring platforms of ITS. The motion state information of each vehicle includes the position, velocity, acceleration, etc. The monitoring platform can also use the estimated states to do such tasks as trajectory planning of vehicles or adjusting traffic scene and so on. However, the wireless communication of IoV is vulnerable to be attacked due to its openness. Thus, the vehicle states that the monitoring platform received may be corrupted by special cyber-attackers. Aiming at the problem, we now establish the CACC models of the connected vehicle system and the false data injection attack models.

### 2.1. CACC Models for Connected Vehicles

**Figure 1:**
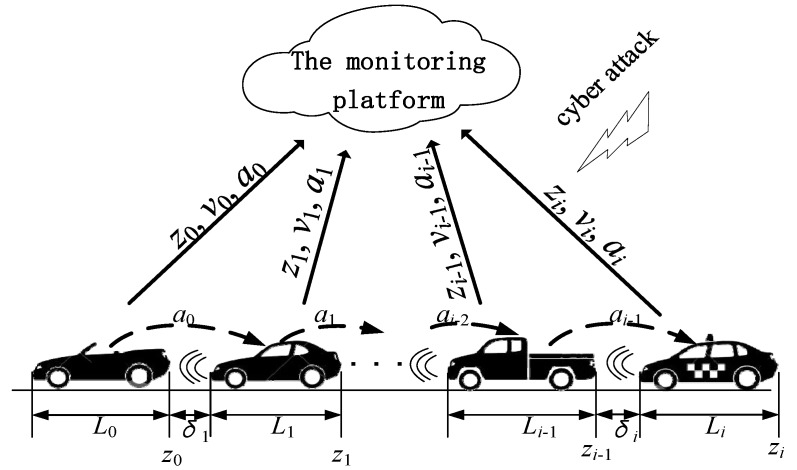
A schematic of intelligent connected vehicle systems with cooperative adaptive cruise control (CACC) and cyber-attack.

Consider a CACC system of connected and networked vehicles, as shown in Figure 1. 

There are *n* + 1 vehicles running on a single lane being level with no effects of the wind speed. In this paper, we assume that the leading vehicle (*i* = 0) is running at a constant speed. For each vehicle *i* = 1,…, *n*, the desired spacing error is defined as:

(1) $$\delta_{i}=z_{i-1}-z_{i}-\delta_{d}-L_{i}$$

where *δ_i_* is the desired spacing error, *z_i_* and *L_i_* separately represent the absolute position and length of the *i*th vehicle and *δ_d_* is the desired safe inter-vehicle distance (spacing).

For each vehicle *i* = 1,…, *n*, the longitudinal dynamics is described by [10,11]:

(2) $$m_{i}a_{i}(t)=F_{i}(t)-\sigma A_{i}c_{di}v_{i}^{2}(t)/2-d_{mi}$$

where *m_i_* represents the mass of the vehicle, *a_i_* represents the acceleration, *F_i_* is the driving force, σ is the density of air quality, *A_i_* is the windshield area, *c_di_* is the resistance coefficient, *v_i_* is the velocity, *d_mi_* is the mechanical resistance. Moreover, due to focusing on the CACC system, in this paper the vehicular throttle and braking pedal units are assumed to have desired dynamics [11], which is:

(3) $${\dot{F}}_{i}(t)=\left(-F_{i}(t)+c_{i}(t)\right)/\tau_{i}$$

where *τ_i_* > 0 is the constant lag time of the internal actuator dynamics and *c_i_* is the input of the throttle or pedal of the *i*th vehicle. Substituting (2) into (3), we have that:

(4) $${\dot{F}}_{i}(t)=-\frac{1}{\tau_{i}}\left(m_{i}a_{i}(t)+\frac{\sigma A_{i}c_{di}}{2}v_{i}^{2}(t)+d_{mi}\right)+\frac{c_{i}(t)}{\tau_{i}}$$

Dividing *m_i_* on both sides of (4) and then substituting it into the derivative of Equation (2), it is obtained the dynamics of the acceleration variable of the *i*th vehicle is:

(5) $${\dot{a}}_{i}(t)=-\frac{1}{\tau_{i}}\left(a_{i}(t)+\frac{\sigma A_{i}c_{di}}{2m_{i}}v_{i}^{2}(t)+\frac{d_{mi}}{m_{i}}\right)-\frac{\sigma A_{i}c_{di}v_{i}(t)a_{i}(t)}{m_{i}}+\frac{c_{i}(t)}{\tau_{i}m_{i}}$$

For the nonlinear Equation (5), the feedback linearization controller is designed as:

(6) $$c_{i}(t)=\frac{1}{m_{i}\tau_{i}}\left[u_{i}(t)-q\left(v_{i}(t),a_{i}(t)\right)\right]$$

where *u_i_* is the CACC input to be calculated by using the desired spacing error, relative velocity and acceleration between the host vehicle and the front one, and nonlinear term *q*(*v_i_*,*a_i_*) is:

(7) $$q\left(v_{i},a_{i}\right)=-\frac{1}{\tau_{i}}(a_{i}+\frac{\sigma A_{i}c_{di}}{2m_{i}}v_{i}^{2}+\frac{d_{mi}}{m_{i}})-\frac{\sigma A_{i}c_{di}v_{i}a_{i}}{m_{i}}$$

Then the kinematics equation of the *i*th vehicle can be represented as:

(8) $$\left[\begin{array}{l} {\dot{z}}_{i}(t)\\ {\dot{v}}_{i}(t)\\ {\dot{a}}_{i}(t) \end{array}\right]=\left[\begin{array}{ccc} 0 & 1 & 0\\ 0 & 0 & 1\\ 0 & 0 & -1/\tau_{i} \end{array}\right]\left[\begin{array}{c} z_{i}(t)\\ v_{i}(t)\\ a_{i}(t) \end{array}\right]+\left[\begin{array}{c} 0\\ 0\\ 1/\tau_{i} \end{array}\right]u_{i}(t).$$

In this paper, the CACC controller of each vehicle *i* = 1,…,*n* is designed as an output feedback Proportion-Differentiation (PD) controller:

(9) $$u_{i}(t)=K_{i}y_{i}(t),\;t\geq 0$$

where the controller gain *K_i_* = [*k_z_*,*_i_*, *k_v_*,*_i_*, *k_a_*,*_i_*] and the output vector *y_i_* = [*z_i_*_−1_−*z_i_*, *v_i_*_−1_−*v_i_*, *a_i_*_−1_−*a_i_*]^T^. In CACC systems, PD controllers are the widely used due to simplicity and efficiencies [12,13,14,15,16,17]. Here the gain *K_i_* is assumed to be calculated using the spired spacing error, relative velocity and relative acceleration to ensure the string stability of the CACC system [13]. In CACC, the output *y_i_* can be measured by the onboard sensors, e.g., radars, Lidar, etc., and the acceleration of the front vehicle is transmitted by wireless IoV communication.

We stack the state vector of the connected vehicle platoon, i.e., *x* = [*z*_1_, *v*_1_, *a*_1_,⋅⋅⋅, *z_i_*, *v_i_*, *a_i_*,⋅⋅⋅, *z_n_*, *v_n_*, *a_n_*]^T^. Then the closed-loop CACC system of the connected vehicle platoon has the compact form of:

(10) $$\dot{x}(t)=\hat{A}x(t)+\hat{G},\;t\geq 0$$

where the matrices are:

$$\hat{A}=\left[\begin{array}{ccccc} D & 0 & \cdots & 0 & 0\\ H_{1} & 0 & \cdots & 0 & 0\\ 0 & D & \cdots & 0 & 0\\ H_{2}^{\prime } & H_{2} & \cdots & 0 & 0\\ \vdots & \vdots & \ddots & \vdots & \vdots \\ 0 & 0 & \cdots & 0 & D\\ 0 & 0 & \cdots & H_{n}^{\prime } & H_{n} \end{array}\right],\;\hat{G}=\left[\begin{array}{c} 0\\ H_{1}^{\prime }{\left[\begin{array}{ccc} z_{0} & v_{0} & a_{0} \end{array}\right]}^{T}\\ 0\\ 0\\ 0\\ \vdots \\ 0 \end{array}\right],$$

$$\left\{ \begin{array}{l} H_{i}=\left[\begin{array}{ccc} -k_{z,i}/\tau_{i} & -k_{v,i}/\tau_{i} & -(k_{z,i}+1)/\tau_{i} \end{array}\right]\\ H_{i}^{’}=\left[\begin{array}{ccc} k_{z,i}/\tau_{i} & k_{v,i}/\tau_{i} & k_{a,i}/\tau_{i} \end{array}\right] \end{array}\right.,\;D=\left[\begin{array}{ccc} 0 & 1 & 0\\ 0 & 0 & 1 \end{array}\right]$$

for *i* = 1, …, *n*. In order to securely estimate the motion states of the vehicle CACC system (10) by the sampled output measurements, the CACC system is discretized with a sampling time *T* > 0. Namely, the discrete-time state space model of the closed-loop CACC system of the connected vehicle platoon is represented as:

(11) x(k+1)=Ax(k)+G(k), k=0,1,2⋯

where matrices *A* = *E* + *ĀT*, *G* = *ĜT*, and *E* is an identity matrix with appropriate dimension.

### 2.2. False Data Injection Attack Models

In this paper, we consider the class of cyber-attack which is occurred in the communication layer linking the vehicles with the monitoring platform. The wireless channels, which deliver the vehicle’s state information from the CACC system to the monitoring platform, are attacked by cyber attackers (see Figure 1). In this way, the attackers can cheat the monitoring platform by tampering the data of the motion states of the vehicles in the intelligent connected vehicle system. Consequently, the monitors in the remote monitoring platform may make wrong decisions.

There are many kinds of cyber-attack such as denial of service (DoS), interference attack, false data injection attack and so on [21,22,23]. In this section we consider the false data injection attack. The attacker firstly attacks the wireless networks through truncating the package of the cruise states and modifying the payload, and then delivers the corrupted package to the monitoring platform. This may cause the monitoring platform to make incorrect judgments about the real operation of the CACC system and ultimately interfere with the normal operation of the monitoring platform.

In this scenario, every vehicle of the vehicle CACC system delivers the information such as absolute position, velocity and acceleration to the remote monitoring platform. Once the data package is in the communication layer linking the vehicles with the monitoring platform, the related data are modified by cyber attackers. It has been shown that the real cruise states cannot be reconstructed if the attacked node (state) is more than the half of the total quantity [30,31]. Hence, here we assume that the attacked date is no more than half of the total quantity of the state of the connected vehicle platoon at each time instant. The assumption is reasonable because the malicious attackers always want to be hidden and their abilities are limited by the economic capability. In other words, there is no way to reconstruct the motion states of vehicles if an attacker has an ability to truncate all packages transmitted to the monitoring networks, and even the attacker can simulate any vehicles running scenarios but cannot be detected.

Now we establish the false data injection attack model of the vehicle CACC system (11). From the viewpoint of the monitoring platform, the vehicle’s state space model is selected as (11). If the wireless channels linking the vehicles with the monitoring platform are not attacked and the data is received correctly by the monitoring platform, then the data is obtained by:

(12) y(k)=Cx(k)

where *y*(*k*) is the received data on the monitoring platform, and *C* is the observation matrix being identity matrix with appropriate dimension.

However, if the false data injection attack is occurred, the received data *y*(*k*) will be introduced an unknown value compared to the actual states. For the monitoring platform, the attacked values are added to the state which are then delivered through the wireless channels. Hence, we present the false data injection attack model as:

(13) y(k)=Cx(k)+Γe(k)

where Γ = diag(*λ*_1_,···, *λ_t_*) represents the attack selection matrix, the *i*th data is attacked if *λ_i_* = 1; otherwise, *λ_i_* = 0, and the signal *e*(*k*) represents the attack values injected to the vehicle’s state information which is delivered through the wireless channels to the monitoring platform.

## 3. Secure State Estimation 

In order to reconstruct the initial states of the vehicle CACC system (11), let we first consider the principle of compressive sensing [32]:

(14) $$\underset{x}{\min}{\left\|x\right\|}_{0}\;s.t.\;b=Px$$

where *b* ∈ *R^m^* is the measurements, *P* ∈ *R^m^*^×^*^n^* is a sensing matrix and ‖*x‖*_0_ denotes the number of nonzero elements for the vector *x*. If the sparse vector *x* meets ‖*x‖*_0_ = *q* ≤ *m*/2 and all subsets of 2*q* columns of *P* are full rank, then the solution to (14) is unique [32].

To reconstruct the states attacked through the above compression sensing method, we integrates the attacked CACC system of the connected vehicles described by (11)–(13) as:

(15) $$\left\{ \begin{array}{l} x(k+1)=Ax(k)+G(k)\\ y(k)=Cx(k)+\Gamma e(k) \end{array}\right.$$

where the diagonal matrix Γ corresponds with the data package which is under attacking.

To solve the problem of reconstructing the state at the initial time using the compressive sensing method, now we consider the set of output measured values *y*(*k*), *k* = 0, …, *M* − 1, which are destroyed at successive *M* times. From the model (15), we stacked the *M* output measurements as:

(16) $$Y={\left[\begin{array}{cccc} y(0) & y(1) & \cdots & y(M-1) \end{array}\right]}^{T}=\Phi x(0)+E+\hat{Y}$$

where the coefficient matrices are:

$$\Phi =\left[\begin{array}{c} C\\ CA\\ \vdots \\ CA^{M-1} \end{array}\right],\;E=\left[\begin{array}{c} \Gamma e(0)\\ \Gamma e(1)\\ \vdots \\ \Gamma e(M-1) \end{array}\right],\;\hat{Y}=\left[\begin{array}{cccc} 0 & 0 & \cdots & 0\\ C & 0 & \cdots & 0\\ \vdots & \vdots & \ddots & \vdots \\ CA^{M-2} & CA^{M-3} & \cdots & 0 \end{array}\right]\left[\begin{array}{c} G(0)\\ G(1)\\ \vdots \\ G(M-1) \end{array}\right].$$

Moving the term *Ŷ* to the left of equation (16), we have:

(17) $$\bar{Y}=\Phi x(0)+E$$

where $\bar{Y}=Y-\hat{Y}$.

In order to resolve the problem of the state estimation, we should determine the value of *M*. From the results in [30,31], the value of *M* is equal to the number of states if the attacked states are fixed; but if the attacked states are varying, *M* may be greater than the number of states in some cases. Through the simulation experiments (see later), under the condition of varying attacked states, the states also can be reconstructed successfully if *M* is equal to the number of the states. As a result, we can estimate the error vector *E* firstly and next we reconstruct the initial state *x*(0) through the estimated value of *E*.

In order to achieve the purpose of estimating *E*, we use the orthogonal decomposition to the matrix *Φ* ∈ *R^pM^*^×3^*^n^*, where *p*, *M* and *n* represents the number of the sensors, measurements and vehicles of the CACC system, respectively. Consider the orthogonal decomposition of *Φ* as:

(18) $$\Phi =\left[\begin{array}{cc} Q_{1} & Q_{2} \end{array}\right]\left[\begin{array}{c} R_{1}\\ 0 \end{array}\right]$$

where [*Q*_1_ *Q*_2_] is an orthogonal matrix with *Q*_1_ ∈ *R^pM^*^×3^*^n^* and *Q*_2_ ∈ *R ^pM^*^×^^(*pM*^^−^^3*n*)^ and *R*_1_ ∈ *R*^3*n*×3*n*^ is an upper triangular matrix with full rank.

Substituting Equation (18) into Equation (17), we can obtain that:

(19) $$\bar{Y}=\left[\begin{array}{cc} Q_{1} & Q_{2} \end{array}\right]\left[\begin{array}{c} R_{1}\\ 0 \end{array}\right]x(0)+E.$$

Because the matrix [*Q*_1_ *Q*_2_] is the orthogonal matrix. Multiplying the matrix [*Q*_1_ *Q*_2_]^T^ on both sides of (19), it is obtained that:

(20) $$\left[\begin{array}{c} Q_{1}^{T}\\ Q_{2}^{T} \end{array}\right]\bar{Y}=\left[\begin{array}{c} R_{1}\\ 0 \end{array}\right]x(0)+\left[\begin{array}{c} Q_{1}^{T}\\ Q_{2}^{T} \end{array}\right]E.$$

Simplifying (20), it is derived that:

(21) $$Q_{1}^{T}\bar{Y}=R_{1}x(0)+Q_{1}^{T}E$$

(22) $$Q_{2}^{T}\bar{Y}=Q_{2}^{T}E$$

Since the number of attacked states is less than *p*/2, where *p* represents the number of the delivered states at each moment, the solution to Equation (22) is unique from the principle of compressive sensing in Equation (14). Because the intelligent connected vehicle system consists of *n* vehicles with together the leader vehicle and every vehicle has three state variables, then the number of the attacked states are up to ⌊3n/2⌋ at each time instant.

Now we use the compressive sensing method to estimate the attack vector *E* by solving the following optimization problem:

(23) $$\hat{E}=\underset{E}{\min}{\left\|E\right\|}_{L}{}_{{}_{0}}\;s.t.\;Q_{2}^{T}\bar{Y}=Q_{2}^{T}E.$$

Note that the solution of Equation (23) involves the *L*_0_ norm but the *L*_0_ norm optimization is an NP hard problem. As a result, the computational burden of solving Equation (23) is too heavy to efficiently solve the problem. To this end, we can transform the *L*_0_ norm optimization to *L*_1_ norm optimization as the *L*_1_ norm is the optimal convex approximation of the *L*_0_ norm:

(24) $$\hat{E}=\underset{E}{\min}{\left\|E\right\|}_{L1}\;s.t.\;Q_{2}^{T}\bar{Y}=Q_{2}^{T}E.$$

We can obtain the estimation *Ê* by computing Equation (24). Clearly, this approximation greatly reduces the computing burden of solving the estimation of the attack vector *E*. Moreover, substituting *Ê* into (21) and simplifying the equation, we derive the initial state *x*(0) by:

(25) $$x(0)=R_{1}^{-1}Q_{1}^{T}(\bar{Y}-\hat{E}).$$

Then the actual motion states of each vehicle in the intelligent connected vehicle system can be evaluated real-time by iterative computing Equation (11).

It is noted that from the compressive sensing method [30,31], it is ensured that the solution of *Ê* is unique. Because the matrix *R*_1_ is an upper triangular matrix with full rank, the initial state *x*(0) is also unique. Therefore, in the remote monitoring platform the presented secure state estimate method is used to real-time obtain the actual motion states of each vehicle in the intelligent connected vehicle system even under cyber-attack of false data injection.

## 4. Simulation Results

In this section, we show the validity of the presented secure state estimate method of the intelligent connected vehicle system. The simulation scene here considers a group of four heterogeneous vehicles running in a single lane, where all vehicles are equipped with the PD-type CACC controllers. Moreover, the vehicle CACC system is stable and string stable. There is the remote monitoring layer which monitors the vehicular motion states through wireless IoV communication (see Figure 1). The wireless channels may be maliciously attacked by the false data injection from cyber attackers. Using the presented secure state estimate method to recover the corrupted received data, the remote monitoring platform can achieve normal operation.

In this simulation study, the vehicles’ parameters are selected as *L_i_* = 4 m for *i* = 0, 1, 2, 3, *τ*_0_ = 0.20 s, *τ*_1_ = 0.25 s, *τ*_2_ = 0.20 s, *τ*_3_ = 0.25 s and *δ_d_* = 2 m. Note that the subscript “0” represents the leading vehicle and the others represent the following vehicles. For the wireless channels which link the vehicles to the remote monitoring layer, the observation matrix *C* is chosen as a nine-order identity. Moreover, the PD-type CACC controllers are calculated by the method in [12] and the controllers’ gains are selected as *K*_1_ = [0.2284, 0.7740, 0.1961], *K*_2_ = [0.2181, 0.7456, 0.1466] and *K*_3_ = [0.2360, 0.8084, 0.2280]. In addition, the simulation scenario is initialized such the case that the leading vehicle is running at the position of 40 m with the velocity of 20 m/s. Because the leading vehicle is running with the constant velocity, the motion state-space model of the leading vehicle is given as:

(26) $$\left[\begin{array}{c} {\dot{z}}_{0}(t)\\ {\dot{v}}_{0}(t)\\ {\dot{a}}_{0}(t) \end{array}\right]=\left[\begin{array}{ccc} 0 & 1 & 0\\ 0 & 0 & 1\\ 0 & 0 & 0 \end{array}\right]\left[\begin{array}{c} z_{0}(t)\\ v_{0}(t)\\ a_{0}(t) \end{array}\right]$$

where *z*_0_, *v*_0_ and *a*_0_ represents the absolute position, velocity and acceleration of the leading vehicle. The matrix *G* in (11) is calculated through the leading vehicle’s states. At the initial time instant, the three following vehicles stop at the position of 25 m, 10 m and 0 m, and the velocity and acceleration are zero, that is, *x*(0) = [25, 0, 0, 10, 0, 0, 0, 0, 0]^T^. Because the number of the states of the vehicle CACC system is 9, then the number of measurements of the system is selected at least as *M* = 9.

**Figure 2:**
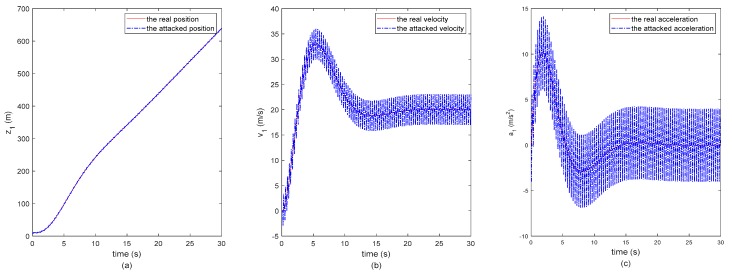
The second following-vehicle’s real states and attacked states. (**a**) The absolute position, (**b**) The velocity, (**c**) The acceleration.

**Figure 3:**
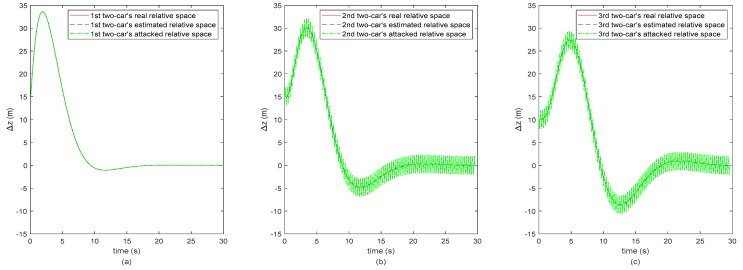
The three 2-cars’ spacing. (**a**) The 1st two-car, (**b**) The 2nd two-car, (**c**) The 3rd two-car.

**Figure 4:**
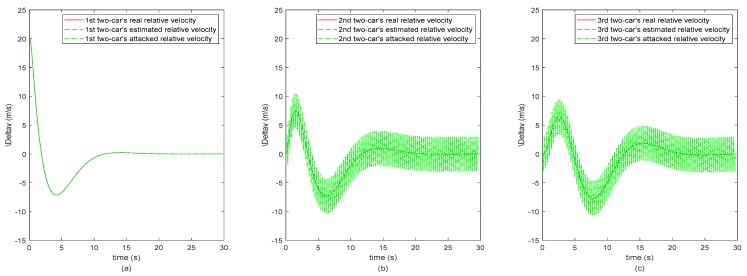
The three 2-cars’ relative velocity. (**a**) The 1st two-car, (**b**) The 2nd two-car, (**c**) The 3rd two-car.

**Figure 5:**
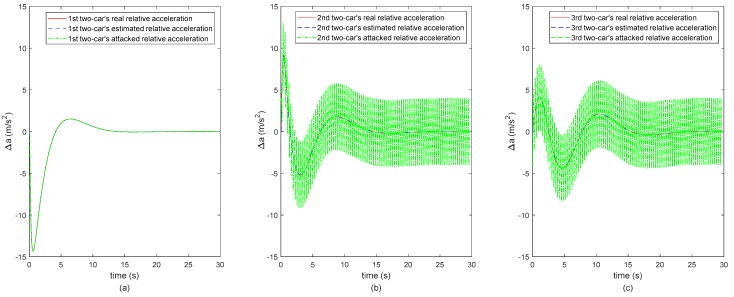
The three 2-cars’ relative acceleration. (**a**) The 1st two-car, (**b**) The 2nd two-car, (**c**) The 3rd two-car.

In the simulation experiment, it is assumed that attackers want to maliciously interfere with the normal operation of the remote monitoring platform of the intelligent connected vehicle system. Hence, they randomly attack the data packets in the remote monitoring networks. In this scene, it is assumed that the second following-vehicle is under attacking. The data delivered to the monitoring platform is injected by the false data from malicious attackers, which is shown in Figure 2. Note that this attack is launched randomly to the three states of this vehicle in this study. It is observed from Figure 2 that the three states of this vehicle are attacked and the other vehicles’ states are not attacked. In Figure 2, the red dashed and dot-dashed lines represent the real and the attacked states of the following vehicles, respectively. Then we use the presented secure state estimate method to estimate the real states of the vehicle platoon. The secure state estimate results are shown in Figure 3, Figure 4 and Figure 5.

In order to clearly analyze the results on secure state estimation of the vehicle CACC system, we use the spacing, relative velocity and acceleration profiles of the adjacent two vehicles to replace the absolute position, velocity and acceleration profiles. Hence, the subplots (a) of Figure 3, Figure 4 and Figure 5 represent the 1st two-car’s inter-vehicle distance, relative velocity and relative acceleration profiles, respectively, and subplots (b) and (c) represent the 2nd two-cars’ and the 3rd two-cars’, respectively. Note that the second following-vehicle is under attacking and the ranges of attack are as shown in Figure 2. As the CACC system (11) used in the remote monitoring platform is dependent on the inter-vehicle distance, relative velocity and acceleration between adjacent vehicles, the monitoring motion states of the third following-vehicle are also negatively, indirectly affected by the attack launched to the second following-vehicle. Hence, in Figure 3, Figure 4 and Figure 5, the 2nd and 3rd two-cars’ green dot-dashed lines are different from the 1st two-cars’, which represent the values after attacking.

It is observed from Figure 3, Figure 4 and Figure 5 that the red dashed lines and the blue dotted lines almost coincide, where the two sets of lines represent the real states and the estimated motion states of the vehicle CACC system, respectively. The estimated motion states of the vehicle CACC system are calculated from the attacked states by applying the proposed secure state estimation method. In other words, the motion states of the vehicle CACC system are estimated successfully in the context that the states of the second following-vehicle are under attacking randomly. Note that the attack launched here is hidden in the sense that it is intermittent to inject false data to the states of the second following-vehicle and the number of the attacked states is limited and may change over the time. Hence, the simulation results illustrate the effectiveness of the proposed secure state estimation method for remote monitoring of intelligent connected vehicle systems under the false data injection attack. The proposed estimation method increases the resilient ability of the remote monitoring platform of connected vehicles against to cyber-attack.

## 5. Conclusions

In this paper, we considered the false data injection attack on the wireless networks of intelligent connected vehicle systems and presented the secure state estimation method to reconstruct the motion states of the connected and networked vehicles equipped with the CACC systems. Applying the principle of compressed sensing, the optimization-based state estimation method was proposed to reconstruct the initial state of the vehicle. The simulation results demonstrated the effectiveness of the secure state estimation approach for remote monitoring motion of connected vehicles against to the false data injection attack. In the future, distributed secure state estimation with consideration of process and measurement noises is the pursuing work in order to more effectively reconstruct the motion states of connected vehicles against to cyber-attacks.
